# Critical role of NF-κB in pancreatic cancer

**DOI:** 10.18632/oncotarget.2624

**Published:** 2014-12-10

**Authors:** Lakshmi Prabhu, Rasika Mundade, Murray Korc, Patrick J. Loehrer, Tao Lu

**Affiliations:** ^1^ Department of Pharmacology and Toxicology, Indiana University School of Medicine, Indianapolis, IN, USA; ^2^ Department of Medicine, Indiana University School of Medicine, Indianapolis, IN, USA; ^3^ Department of Biochemistry and Molecular Biology, Indiana University School of Medicine, Indianapolis, IN, USA; ^4^ Division of Hematology and Oncology, Indiana Cancer Pavilion, Indianapolis, IN, USA; ^5^ Department of Medical and Molecular Genetics, Indiana University School of Medicine, Indianapolis, IN, USA

**Keywords:** NF-κB, pancreatic cancer

## Abstract

Pancreatic ductal adenocarcinoma (PDAC) is one of the deadliest cancers, and in spite of intense efforts there are limited therapeutic options for patients with PDAC. PDACs harbor a high frequency of Kras mutations and other driver mutations that lead to altered signaling pathways and contribute to therapeutic resistance. Importantly, constitutive activation of nuclear factor κB (NF-κB) is frequently observed in PDAC. An increasing body of evidence suggests that both classical and non-classical NF-κB pathways play a crucial role in PDAC development and progression. In this review, we update the most recent advances regarding different aspects of NF-κB involvement in PDAC development and progression, emphasizing its potential as a therapeutic target and the need to discover pathway-specific cytosolic NF-κB regulators which could be used to design novel therapeutic strategies for PDAC.

## INTRODUCTION

### NF-κB superfamily and its signaling pathways

The nuclear factor κB (NF-κB) transcription factor was discovered when it was observed that it binds to the enhancer element of the immunoglobulin κ light-chain of activated B cells [1]. NF-κB plays critical roles in inflammation, cell proliferation and differentiation, immune response and cancer [2]. There are five proteins in the mammalian NF-κB family: RelA (p65), RelB, c-Rel, p50/p105 and p52/p100. They all share a Rel homology domain (RHD) in their N-terminus, which results in their classification as NF-κB/Rel proteins. The RHD is essential for dimerization as well as binding to κB cognate DNA elements. NF-κB activity is primarily regulated by interaction with a family of inhibitors called IκBs (inhibitor of κB) proteins. IκB proteins mask the nuclear localization signals (NLS) of NF-κB proteins and keep them sequestered in a latent, inactive complex in the cytoplasm.

There are two different pathways involving NF-κB: the classical (or canonical) and non-classical (or non-canonical) pathways. In the classical NF-κB activation pathway, upon receiving extracellular signals such as stress, cytokines, free radicals, or radiation, the IκB kinase (IKK) phosphorylates IκB [3], which then signals the ubiquitination and degradation of IκB by the proteasome system. The p65/p50 heterodimer of NF-κB is then released and can rapidly enter the nucleus to ‘turn on’ the expression of a variety of κB-dependent genes. By contrast, the non-classical pathway is mediated via p100/RelB complexes that are normally inactive in the cytoplasm. Signaling through a subset of receptors, such as LTβR (lymphotoxin β receptor) and CD40, activates the NF-κB-inducing kinase (NIK), which in turn activates IKKα complexes that phosphorylate C-terminal residues in p100. Phosphorylation of p100 leads to its ubiquitination and proteasomal processing to p52, leading to the formation of p52/RelB complexes that translocate to the nucleus and induce target gene expression.

### Correlation between NF-κB with different types of cancer

Constitutive NF-κB activation has been noted in 95% of all cancers [4–6]. Oncogenic roles of NF-κB include promotion of cell proliferation, control of apoptosis, and stimulation of angiogenesis, invasion/metastasis in cancer cells.

Numerous studies have now documented the role of NF-κB in the development of solid malignancies. For instance, NF-κB has been shown to be involved in the development of breast cancer. Thus, constitutive NF-κB DNA-binding activity is seen in mammary carcinoma cell lines and primary breast cancer cells of human and rodent origin [7]. Furthermore, NF-κB has been shown to function as a key link between inflammatory bowel disease (IBD) and colorectal cancer (CRC) by regulating inflammatory responses and providing a barrier against extrinsic hazard. Studies with azoxymethane (AOM)/dextran sodium sulfate (DSS) mouse models of colitis-associated colon cancer (CAC) and animal models carrying a conditional disruption of IKKβ have been used to show that IKKβ-driven NF-κB activation within intestinal epithelial cells (IECs) is essential for CRC development [8–10]. Another example is the involvement of NF-κB in melanoma, the most aggressive form of skin carcinoma [11]. It has been demonstrated that upregulation of the NF-κB levels is responsible for both the development and metastasis of melanoma. In addition to solid tumors, since NF-κB plays a very important role in immune cell function, hyperactive NF-κB is also frequently associated with the development of leukemia and lymphoma [12].

### Role of NF-κB in pancreatic ductal adenocarcinoma (PDAC)

It is generally considered that oncogenic *Kras* is the initiating molecular alteration in PDAC [13–15]. Its importance in PDAC became clearly evident with the generation of the first genetically engineered mouse model (GEMM) of PDAC that truly recapitulated the histological features seen in humans. Thus, *KC* (stands for Kras;Pdx1-Cre recombinase) mouse carries an oncogenic *Kras* (*Kras*^G12D^) allele that has been knocked-in within its own locus and silenced by the insertion of an upstream LoxP-Stop-LoxP (LSL) element [16]. Oncogenic *Kras* transcription is thus controlled by its endogenous promoter, and the transcript is expressed in early pancreatic progenitors after LSL excision by a pancreas-specific Cre recombinase such as Pdx-1. *KC* mice develop low grade pancreatic intra-epithelial neoplasia (PanIN) and exhibit areas of acinar to ductal metaplasia (ADM) by 2 months of age [16]. By age 8 to 12 months, *KC* mice develop PDAC at moderate penetrance [16]. PanIN is a crucial feature of PDAC initiation in humans and GEMMs, and PanIN progression to PDAC is accelerated by loss of tumor suppressor genes such as *p53*, *Cdkn2A (*cyclin-dependent kinase inhibitor 2A), *Smad4* (mothers against decapentaplegic homolog 4), *Pten* (phosphatase and tensin homolog), or *Rb* (retinoblastoma protein) [17–21].

PDAC exhibits marked resistance to chemotherapy and radiotherapy, a propensity to metastasize, and a dismal 5-year survival rate of 6% [22–24]. Given the role of NF-κB in different cancer types, and the fact that NF-κB transcriptional factors are constitutively activated in the majority of PDACs, it is likely that NF-κB-driven pathways are involved in the regulation of numerous aspects of PDAC development and progression.

### Links between Kras mutation and NF-κB signaling in PDAC

**Figure 1:**
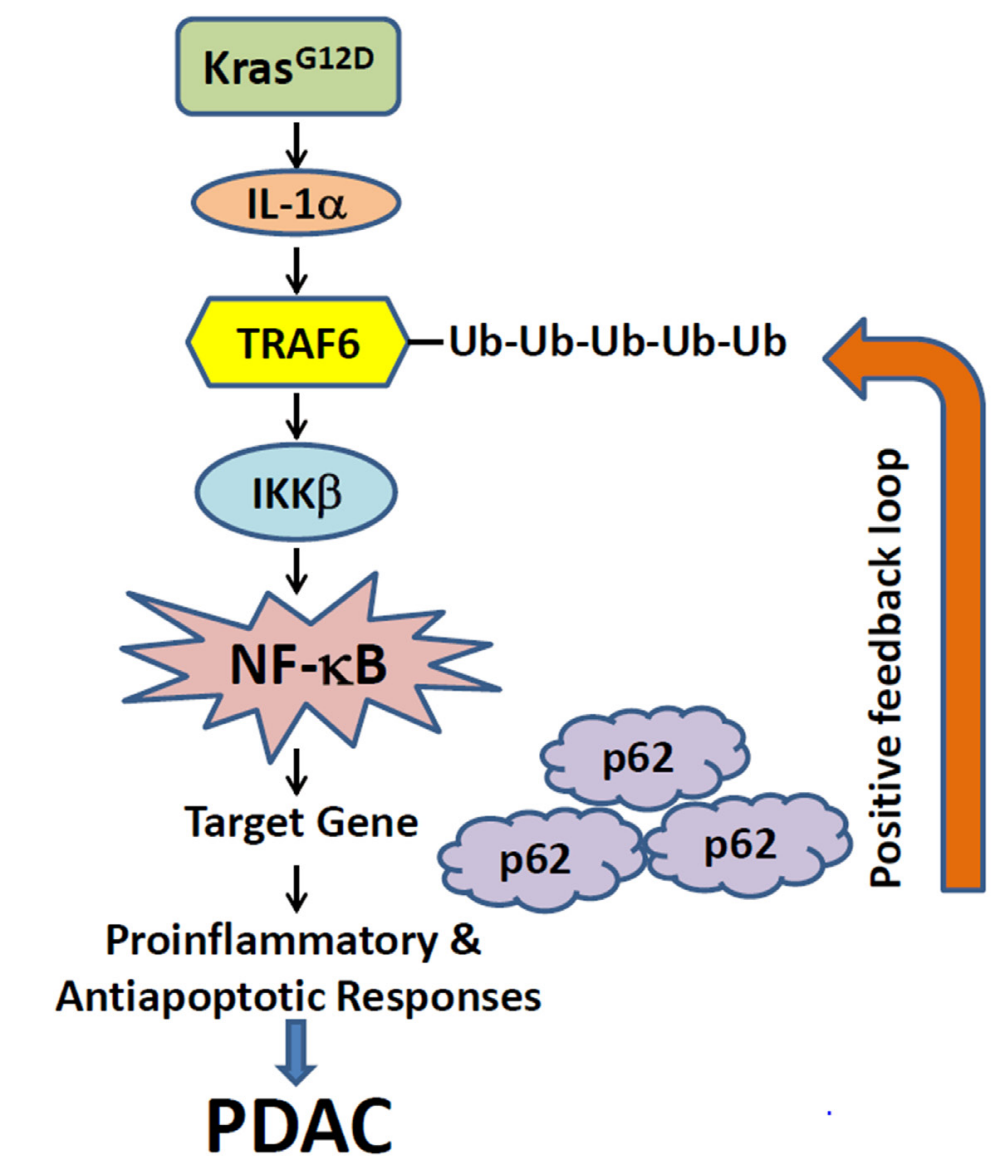
A proposed working model illustrates the potential mechanism through which KrasG12D oncogenic signaling induces positive feedback loops of IL-1α and p62 to sustain constitutive IKKβ/NF-κB activation in PDAC development KrasG12D induces the secretion of IL-1α, which further leads to the ubiquitination of TRAF6 and activation of IKKβ. This would further activate NF-κB to induce its target genes, including *p62*. The p62 protein in turn will positively regulate TRAF6 ubiquitination and lead to the constitutive NF-κB activation and PDAC development (Adapted from Ref. 26).

NF-κB is known to be constitutively activated in most PDAC patients [25]. Instances of NF-κB pathway interacting with concurrent signaling pathways have been recently documented, which might contribute towards the PDAC etiology. For instance, it was shown that the *Kras^G12D^* mutation seen in almost all PDAC cases is the main driver of constitutive NF-κB expression in PanINs and PDACs. As shown in Figure 1, the *Kras* mutation correlates with overexpression of interleukin-1α (IL-1α), a cytokine that binds to its receptor on the cell surface, through activation of AP-1. AP-1 is a transcription factor that is upregulated in *Kras* mutated cells and drives IL-α transcription. This leads to the polyubiquitination of tumor necrosis factor (TNF) receptor-associated factor 6 (TRAF6) at lysine 63 (K63). The K63-ubiquitin-TRAF6 can be recognized by the K63-ubiquitin-binding activity of NEMO [a component of the IκB kinase (IKK) complex], therefore, recruiting IKK to the cellular surface. This binding activates the IKK complex, which then causes proteasomal degradation of the IκB complex via phosphorylation of IκB. This leads to translocation of NF-κB to the nucleus and activation of its target genes. One of its target genes is *p62*, an adaptor for regulating the turnover of K63-polyubiquitinated proteins. Thus, this generates an autoregulatory loop where NF-κB upregulates *p62* expression, which in turn helps to drive NF-κB activity. Thus, IL-1α acts as a link between the Kras and NF-κB signaling pathways, which act in concert to drive tumor initiation and progression in PDAC [26].

In the context of Kras-induced PDAC, it has also been shown that NF-κB and Notch signaling interact to drive a sustained inflammatory response in transformed cells [27]. As a major transcription factor for these inflammatory responses, NF-κB is found to be activated in Kras-transformed epithelial cells [27]. Its activation further leads to the expression and secretion of many NF-κB responsive inflammatory cytokines, such as TNF-α. This in turn binds to its receptor to activate the NF-κB pathway through the IKK complex (comprising of IKK1, 2 and 3 subunits) via the classical NF-κB activation pathway. In collaboration with basal Notch signals, NF-κB activation induced optimal expression of Notch-regulated genes *Hes1* (hairy and enhancer of split-1) and *Hey1* (Hairy/enhancer-of-split related with YRPW motif protein 1). Mechanistically, TNF-α stimulation resulted in phosphorylation of histone H3 at the *Hes1* promoter, and this signal was lost with *Ikk2* gene deletion. Hes1 suppresses expression of the anti-inflammatory nuclear receptor Pparγ (Peroxisome proliferator-activated receptor gamma). Thus, crosstalk between TNF-α/IKK2 and Notch sustains the intrinsic inflammatory profile of transformed cells. These findings reveal a novel interaction between oncogenic inflammation and a major cell fate pathway and show how Kras, NF-κB and Notch pathways can cooperate to promote cancer progression in PDAC cells.

### NF-κB and cytokine/chemokine secretion in PDAC

In addition to its role in the Kras-induced PDAC, NF-κB also functions as a key link between pancreatic inflammation and cancer. Chronic pancreatitis is a chronic inflammatory condition in which the pancreas harbor inflammatory cells and macrophages, and exhibits enhanced stroma formation. During their lifetime, patients with chronic pancreatitis exhibit a 16-fold increased risk for developing PDAC [23]. One explanation for this increased propensity to develop PDAC is increased cytokine secretion from intra-pancreatic macrophages. Storz and colleagues showed that macrophages from patients with chronic pancreatitis secrete increased amounts of the pro-inflammatory cytokines, RANTES (regulated on activation normal T cell expressed and secreted) and TNF-α, which in turn activate NF-κB and promote expression of NF-κB target genes such as MMP-9 (Matrix metallopeptidase 9). This mechanism has been proposed to lead to ADM and eventually PanIN formation [28]. Another study has also suggested that transforming growth factor β (TGF-β) can activate NF-κB to downregulate the expression of tumor suppressor gene *Pten*, thereby promoting cell motility in PDAC cell lines, thus suggesting that these pathways might get interlinked in PDAC [29]. Interestingly, the TGF-β activated kinase 1 (TAK1) is a key player in the activation of NF-κB in many situations. For example, Shinohara *et al* [30] recently reported that the CARD-containing MAGUK protein 1 (CARMA1)-TAK1-IKKβ module is a switch mechanism for NF-κB activation in B cell receptor (BCR) signaling. Mutation of the scaffolding protein CARMA1 at serine-578, an IKKβ target, abrogated not only late TAK1 activity, but also the activation of NF-κB in B cells, suggesting the intimate connection between TAK1 and NF-κB activation. Previously, we reported that some tumor cell lines secrete high concentrations of TGF-β or IL-1β. We found that similarly high concentrations of each of these cytokines cross-activate the other pathway: TGF-β activates NF-κB, and IL-1β activates the major transcription factor Smads in TGF-β pathway. Importantly, TAK1 is required for this important cross-activation. We also found that cross-talk between the TGF-β and IL-1β signaling pathways leads to dose-dependent cross-control of gene expression. These interactions provide new insight into biological responses to IL-1β and TGF-β in the proximity of tumors that secrete high concentrations of these factors and probably also at sites of inflammation, where the local concentrations of these cytokines are likely to be high [31].

In addition to cytokines, chemokines are also elevated in PDAC cells, and its increase is most likely due to enhanced NF-κB signaling. For instance, CXCL14 is a chemokine that promotes angiogenesis and tumor growth and its levels are elevated in PDAC [32]. Increased CXCL14 levels have been shown to promote translocation of NF-κB to the nucleus, thus activating the NF-κB signaling pathway [32].

### Other factors that affect NF-κB in PDAC

Besides cytokines and chemokines, NF-κB activity can also be affected by other factors, such as microRNAs, posttranslational modifications, and reactive oxygen species (ROS). For example, in normal cells, NF-κB repressing factor (Nrkf) down regulates NF-κB expression. However, in PDAC cells, the activity of Nrkf is down regulated by microRNA miR-301a, whose activity is upregulated in PDAC. This downregulation of Nrkf leads to the activation of NF-κB, which in turn promotes miR-301a transcription, generating a positive feedback loop to increase NF-κB levels. Overall, this contributes to increased tumor formation in mice [33].

Another characteristic observed in PDAC cell lines is global hyper-O-GlcNAcylation, which also relates to NF-κB. A recent study observed that a direct posttranslational O-GlcNAc modification of the NF-κB p65 subunit and of the IKK complex promoted phosphorylation of p65 and nuclear localization and activation of the NF-κB target genes in PC cell lines [34]. Furthermore, ROS have been implicated in tumor development and proliferation in PDAC. RAGE (receptor for advanced glycation endproducts) is a receptor from the immunoglobulin superfamily involved in ROS generation. Activation of NF-κB leads to the upregulation of RAGE expression in PDAC cells [35]. Thus, NF-κB contributes to PC formation by ROS generation via RAGE. Moreover, the role of NF-κB is not only limited to PC tumor itself, it is also found to play a role in PC-related cachexia (or wasting syndrome) [36].

### Non-classical NF-κB pathway in PC

Although the classical NF-κB pathway is the key contributor to PDAC development, there is increasing evidence that non-classical pathway also contributes to PDAC progression. For example, Storz's group suggested that the non-classical NF-κB pathway is a source for the high basal NF-κB activity in PDAC cell lines [37]. They showed that in Panc1 and MiaPaca2 PC cells, increased activity of the p52/RelB NF-κB complex is mediated through stabilization and activation of NF-κB-inducing kinase (NIK). Their group identified proteasomal degradation of TRAF2 as a mechanism by which levels of active NIK are increased in PC cells. Such upregulation of NIK expression and activity levels eventually led to increased proliferation and anchorage-independent growth of PDAC cells [37].

### Perspective

**Figure 2:**
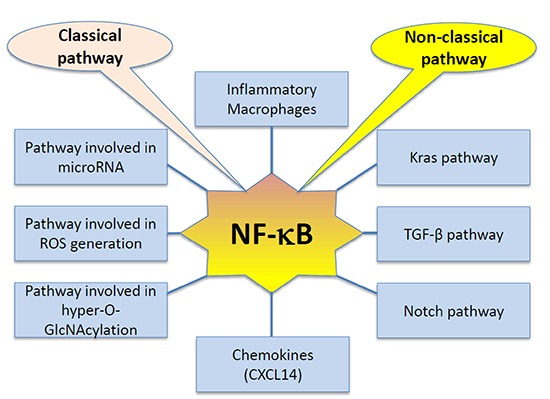
Interactions of NF-κB signaling pathway in pancreatic cancer (PC) As indicated in the diagram, both classical and non-classical NF-κB pathways interact with a number of signaling pathways to remain constitutively activated in pancreatic cancer cells and promotes tumorigenesis and metastasis.

NF-κB pathway is emerging as an important player in PDAC. As illustrated in Figure 2, both the classical and non-classical arms of the pathway have been implicated in promoting disease progression through increased cancer cell proliferation, motility, anti-apoptotic and inflammatory signals that aid in metastasis and disease progression. Not only does the pathway itself contribute to disease initiation and progression, it gets even more complicated since NF-κB interacts with other pathways that are also deregulated in PDAC. This adds to the disruptions in signaling that can be seen in the different PDAC cases.

Numerous inhibitors of the NF-κB pathway have already been described, including small molecules, peptides, small DNA/RNA, viral proteins, natural compounds *etc.* [38]. The majority of these compounds have not been specifically tested in a PDAC model. However, it would be worth testing the efficacy of these compounds as a therapeutic strategy in PDAC. For instance, curcumin, a common cooking spice and resveratrol, found in red grapes and berries have been shown to inhibit the cytokine expression mediated by NF-κB [39]. Curcumin has also been shown to inhibit IKK activity [40]. Compounds derived from flavonoid precursors like chalcones, commonly found in fruits and vegetables were shown to decrease proliferation in human cancer cell lines [41]. Another flavonoid, quercetin interfered with cell cycle progression and induced apoptosis in HeLa cells by inhibiting NF-κB activity [42]. Small molecule inhibitor screenings have also been effective in detecting compounds like N1241, which inhibits the ability of NF-κB to bind to DNA and interferes with its activity [43]. Additionally, drugs like bortezemib and thalidomide, which are approved by the US Food and Drug Administration (FDA) for the treatment of multiple myeloma, may also function, in part, by inhibiting NF-κB activity [44, 45].

Several lines of evidence have suggested that inhibition of NF-κB is effective in PDAC prevention and treatment. For instance, nitric oxide-releasing aspirin (NO-aspirin) represents a novel class of promising chemopreventive agents. Unlike conventional nonsteroidal anti-inflammatory drugs, NO-aspirin seems to be free of adverse effects while retaining the beneficial activities of its parent compound. Recently, Rao *et al.* [46] evaluated the effect of NO-aspirin on pancreatic carcinogenesis in Kras^G12D^ mouse model. Their results revealed that NO-aspirin significantly suppressed pancreatic tumor weight, PDAC incidence, and carcinoma in situ (PanIN-3 lesions) by decreasing ~42% the expression and activity of cyclooxygenase (COX), a renowned NF-κB-dependent gene. Later on, Streicher *et al.* further carried out case-control study of aspirin use and risk of pancreatic cancer [47]. Moreover, since the NF-κB pathway may also contribute to the marked chemoresistance that occurs in PDAC, thus inhibiting NF-κB not only will tackle the problem of PDAC development and progression, but may also attenuate chemoresistance, thereby potentially improving responses to chemotherapy.

PDAC is a complex disease that is all too often therapy-recalcitrant. Our review suggests that combination therapies targeting NF-κB in conjunction with either classical chemotherapy, such as gemcitabine or other targeted therapies could yield promising therapeutic strategies. Direct targeting of NF-κB still faces important challenges due to the vital role that NF-κB plays in normal physiological conditions. In the future, targeting nonredundant cytosolic activators of NF-κB instead of NF-κB itself could represent a better approach to inhibit key processes in PC. In this regard, it makes the discovery of pathway-specific novel activators of NF-κB a very attractive task [48, 49] for drug target identification and PDAC treatment.
